# Assessment of Thigh Angular Velocity by an Activity Monitor to Describe Sit-to-Stand Performance

**DOI:** 10.3390/s22041405

**Published:** 2022-02-11

**Authors:** Jochen Klenk, Alassane Ba, Kim S. Sczuka, Urban Daub, Ulrich Lindemann

**Affiliations:** 1 Department of Clinical Gerontology, Robert-Bosch-Hospital, 70376 Stuttgart, Germany; alassaneba123@gmail.com (A.B.); kim.sczuka@rbk.de (K.S.S.); ulrich.lindemann@rbk.de (U.L.); 2 Institute of Epidemiology and Medical Biometry, Ulm University, 89081 Ulm, Germany; 3 Study Center Stuttgart, IB University of Health and Social Sciences, 70178 Stuttgart, Germany; 4 Biomechatronic Systems, Fraunhofer Institute for Manufacturing Engineering and Automation IPA, 70569 Stuttgart, Germany; urban.daub@ipa.fraunhofer.de

**Keywords:** accelerometer, activity monitor, angular velocity, reliability, sit-to-stand, validity

## Abstract

The assessment of sit-to-stand (STS) performance is highly relevant, especially in older persons, but testing STS performance in the laboratory does not necessarily reflect STS performance in daily life. Therefore, the aim was to validate a wearable sensor-based measure to be used under unsupervised daily life conditions. Since thigh orientation from horizontal to vertical is characteristic for STS movement, peak angular velocity (PAV) of the thigh was chosen as the outcome variable. A total of 20 younger and older healthy persons and geriatric patients (mean age: 55.5 ± 20.8 years; 55% women) with a wide range of STS performance were instructed to stand up from a chair at their usual pace. STS performance was measured by an activity monitor, force plates, and an opto-electronic system. The association between PAV measured by the thigh-worn activity monitor and PAV measured by the opto-electronic system (gold standard) was r = 0.74. The association between PAV measured by the thigh-worn activity monitor and peak power measured by force plate and opto-electronic system was r = 0.76. The Intra-Class Coefficient (ICC) of agreement between the 2 trials was ICC_(A,1)_ = 0.76. In this sample of persons with a wide range of physical performance, PAV as measured by a thigh-worn acceleration sensor was a valid and reliable measure of STS performance.

## 1. Introduction

Standing up from a seated position is a prerequisite for physical mobility, but it is also associated with falls in frail older persons [1] and in community-dwelling older adults [2]. Furthermore, the quality of chair rise performance is associated with muscle mass, and it is one aspect in the construct of sarcopenia [3]. Therefore, the assessment of the sit-to-stand (STS) transfer is one core element of assessing physical performance, especially in older persons.

STS performance is usually assessed in the laboratory as a measure of capacity (performing as fast as possible) under supervised condition by stopwatch [4], by force plates [5], by linear encoders [6], by opto-electronic systems [7], by electromyography [8], or by body-worn sensors [9]. Regarding ecological validity, the assessment of STS performance during daily life activity may provide further insights, because STS capacity measured under supervised conditions does not necessarily reflect daily life performance, which has already been shown for gait speed [10].

For the assessment of STS transfer performance during daily life activity, only body-worn sensors are eligible [11]. The sensor on the body for recognition of STS transfers has been fixed at the wrist [12], sternum [13], lower back [9,14,15], and thigh [16]. Age-related differences of STS duration were shown by using a lower back-worn sensor [15] and were confirmed by a recent study focusing on STS performance in younger and older adults during daily life [14]. This study has investigated duration, acceleration, and smoothness of STS transfers also measured by a lower back-worn sensor.

The sensor fixation at the thigh may be more appropriate because a change of orientation of the thigh from horizontal to vertical is characteristic for the STS transfer and may outperform STS measurement by a sensor worn on the wrist or lower back [17]. It was shown by Pickford et al. that peak angular velocity (PAV) can be derived from a thigh worn 3-axial accelerometer [16]. The measure differentiated between healthy older adults and stroke patients. However, it remains unclear which STS capability was reflected by this measure.

For older persons, the STS power seems to be an important parameter associated with general physical fitness [18], maximum physical performance [19], falls [20,21], thigh muscle volume [22], and disability [23]. Therefore, any outcome parameter assessed during the STS transfer by a body-worn sensor should be associated with STS power. So far, a method to assess STS power during daily life activity with high accuracy is not available. We hypothesize that PAV of the thigh might be associated with STS power and might serve as a surrogate measure for leg muscle power.

The aim of the study was to validate a wearable sensor-based measure of PAV of the thigh to repeatedly assess STS transfers as a measure of quality of the STS performance with strong association to power and the potential to be used under unsupervised long lasting daily life conditions.

## 2. Materials and Methods

### 2.1. Subjects and Design

Regarding age, gender, and physical performance, a wide range of persons’ STS movement pattern was investigated in this cross-sectional study. In a south-western German clinic of geriatric rehabilitation, younger (employees below 60 years), older healthy adults (voluntary workers 60 years or older), and geriatric patients (60 years or older) were recruited. Besides age, inclusion criteria were the ability to perform the STS transfer without personal help and to follow instructions. Exclusion criteria were severe balance and mobility problems (not able to perform the protocol safely; self-report), acute pain (self-report), vision impairment (not able to read newspaper headlines), and terminal illness. The sample size was chosen pragmatically for this proof-of-concept study [24]. The study protocol was approved by the ethical committee of the University of Tuebingen (855/2019BO2), and all participants gave written informed consent before assessment.

### 2.2. Outcome Parameters and Protocol

An activity monitor including a 3-axes accelerometer (activPAL4 micro, PAL Technologies, Glasgow, UK) was fixed at the front of the left mid-thigh. The sample period was 0.05 s (20 Hz). Angle [°] and PAV [°/s] during the STS transfer were calculated as outcome measures based on the calculations as described in Equation (1) and Equation (2), respectively, using 3-axes acceleration (vertical, anterior-posterior, and medio-lateral). The rationale for choosing angular velocity instead of acceleration as the primary outcome measure is that angular velocity can describe the change of orientation of the thigh. In contrast, acceleration rather describes the change of a point estimate.

(1) $$\mathrm{angle}\left(t\right)\;\left[{}^{\circ}\right]={\;\tan}^{-1}\left(\frac{{\mathrm{acc}}_{\mathrm{Vert}}\left(t\right)}{\sqrt{{\mathrm{acc}}_{\mathrm{AP}}{\left(t\right)}^{2}+{\mathrm{acc}}_{\mathrm{ML}}{\left(t\right)}^{2}}}\right)$$

(2) $$\mathrm{angular}\;\mathrm{velocity}\;\left(t\right)\;\left[{}^{\circ}/s\right]=\frac{\mathrm{angle}\left(t\right)-\mathrm{angle}\left(t-1\right)}{\mathrm{sample}\;\mathrm{period}}$$

During STS transfer, the chair and the participants’ feet rested on a force plate (BP 400600-2000, AMTI, Watertown, MA, USA) integrated in the floor, measuring the ground reaction force [N]. Furthermore, reflective markers of an opto-electronic system (Vicon MX+, Vicon Motion Systems, Oxford, UK) were fixed at landmark points representing the participants´ left thigh. PAV of the thigh during the STS transfer was analyzed by Vicon software and was used as the gold-standard measure. The sample frequency of the force plate was downsampled to match the sample frequency of the opto-electronic system (100 Hz). STS peak power [W] was calculated as the maximum, based on the force plate signals including persons´ body weight [25] and opto-electronically derived vertical velocity of the hip marker as described in Equation (3). STS peak power was used to investigate construct validity.

(3) $$P_{\mathrm{Vert}}\;\left(t\right)\;\left[W\right]={\;F}_{\mathrm{Vert}}\;\left(t\right)\;\cdot {\;V}_{\mathrm{Vert}}\;\left(t\right)$$

with P_Vert_ = vertical power [W]; F_Vert_ = vertical force [N]; and V_Vert_ = vertical velocity [m/s].

The participants started from sitting on a chair of standard height (47 cm), which was equipped with armrests. Participants were instructed to stand up from the chair at a comfortable pace, as they do in their daily life. Three STS transfers were conducted. The first trial was considered as a practice trial. Data acquisition started with the second trial, which was used for correlation analysis of the thigh-worn activity monitor with functional tests (construct validity). The second and third trials were used for correlation analysis of the thigh-worn activity monitor with the gold-standard method and with peak power. At last, the second and third trials were used for reliability analysis [26]. The use of armrests was recorded but was not analyzed separately, because the pressure against the armrests could not be recorded separately.

Finally, two functional tests were conducted to assess construct validity. The participants were instructed to perform the 5-chair rise test [4]. The time to stand up from the chair and sit down again 5-times as fast as possible [s] was measured by a stopwatch. Furthermore, habitual gait speed [m/s] was assessed in the laboratory by a stopwatch over a distance of 8 m with 2 additional meters for acceleration and deceleration [27].

### 2.3. Descriptive Variables

Age and gender were asked, and weight and height of all participants were measured. Comorbidity was assessed via a standardized questionnaire [28] asking for 18 diseases and symptoms. Yes/no answers resulted in a (0–18) sum-score. Habitual gait speed was also used as a descriptive measure of general functional performance.

### 2.4. Statistics

Group mean values with 95%-confidence intervals (CI) were used to describe the outcome parameters. Pearson’s coefficient of correlation was used to describe the association between PAV measured by the activity monitor and PAV measured by the opto-electronic system and peak power. Due to outliers in the distribution, Spearman´s coefficient of correlation was used to describe the association between PAV and functional measures (gait speed and chair rise time). For graphical description, the agreement between PAV measured by the thigh-worn sensor and the opto-electronic system was evaluated using Bland-Altman diagrams with limits of agreement. Linear regression analyses were used to consider if age, weight, and height affected the association between PAV and peak STS power. Reliability was assessed by calculating the Intra-Class Coefficient of agreement A,1. Furthermore, we calculated the typical error of measurement (TEM) and the smallest worthwhile effect from pure SD (SWC), according to [29], for small (0.2), moderate (0.6), and large (1.2) effects for peak angular velocity measured by the thigh-worn activity monitor.

## 3. Results

Twenty participants with a mean age of 55.5 years (standard deviation (SD) 20.8 years) were investigated, including 11 women (55%), 11 persons below 60 years, five older community-dwellers (above 60 years), and four geriatric patients (72 to 92 years). The cohort is described in detail in Table 1. Only the four geriatric patients used the armrests during the STS transfer.

Due to technical problems, the force plate and opto-electronic system’s data of 8 out of 40 measurements in total were not available. The lost data were not available for correlation analysis of PAV measured by the activity monitor with the opto-electronic system (gold-standard) and were also not available for correlation analysis of PAV with peak power (construct validity). All data (*n* = 20) were available for correlation analysis of thigh-worn sensor with 5-chair rise time and gait speed (construct validity) and for reliability analysis.

All outcome measures covered a wide range of performance with 36.0°/s to 187.5°/s PAV, 224.8 W to 843.9 W peak power, 5.2 s to 15.9 s to perform 5 repetitive chair-rises, and 0.75 m/s to 2.29 m/s habitual gait speed. All outcome data are presented in detail in Table 2.

**Figure 1:**
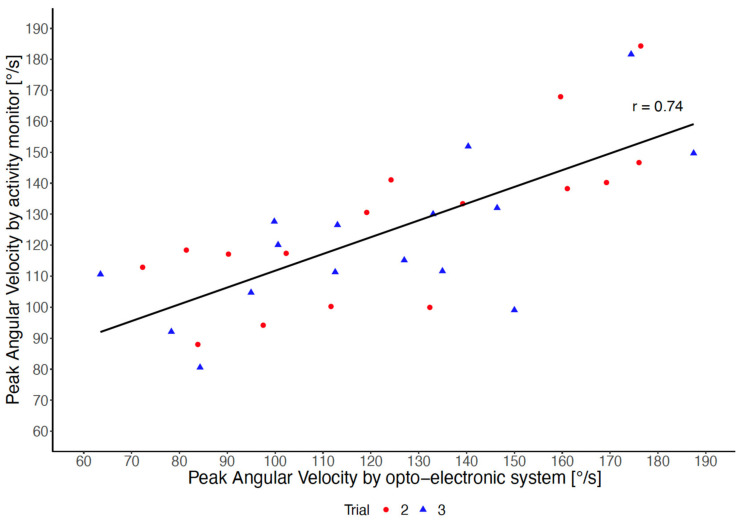
Association between peak angular velocity measured by the thigh-worn activity monitor and peak angular velocity measured by the opto-electronic system, according to Trials 2 and 3.

**Figure 2:**
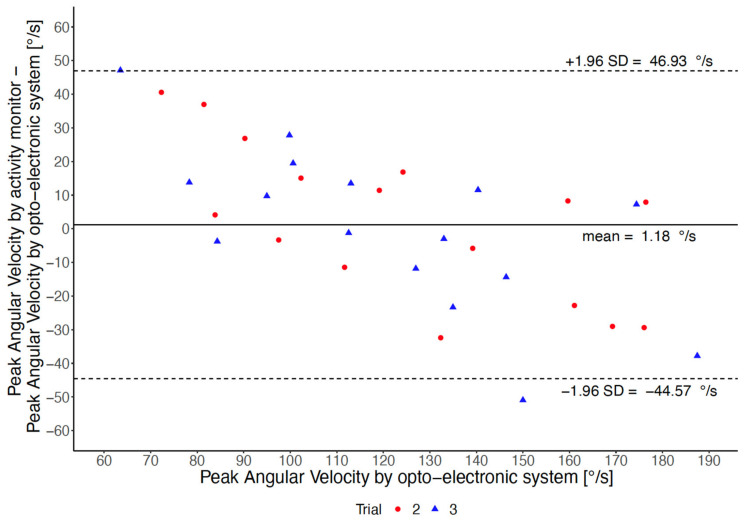
Agreement of peak angular velocity measured by the thigh-worn activity monitor with peak angular velocity measured by the opto-electronic system, according to Trials 2 and 3.

The association between PAV measured by the thigh-worn activity monitor and PAV measured by the opto-electronic system is shown in Figure 1. The Pearson correlation coefficient was r = 0.74, and the intra-class coefficient of agreement was ICC_(A,1)_ = 0.71. The Bland-Altman plot between the two measures is shown in Figure 2.

**Figure 3:**
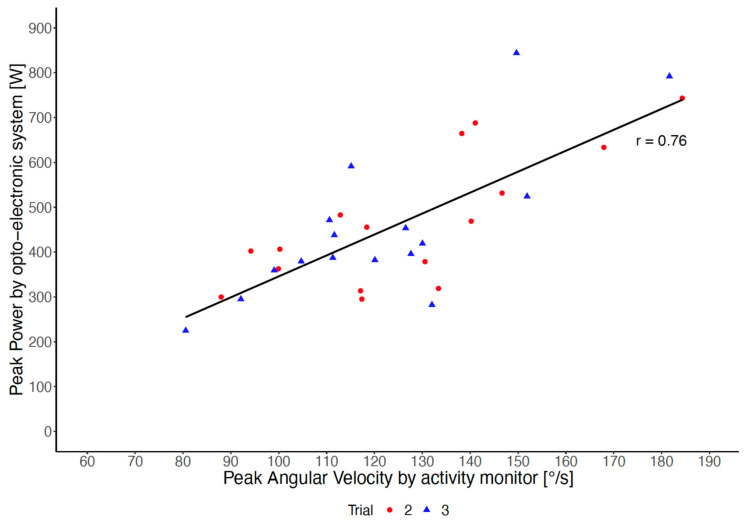
Association between peak angular velocity measured by the thigh-worn activity monitor and peak power measured by the opto-electronic system according to Trials 2 and 3.

The association between PAV measured by the thigh-worn activity monitor and peak power measured by force plates and the opto-electronic system was r = 0.76 corresponding to an adjusted R^2^ of 0.56 (Figure 3). After adjusting angular velocity for body height, the adjusted R^2^ increased. The respective linear regression model explained 73% of variance of peak power during the STS transfer and is shown in Equation (4). Further adjustment for body weight (*p* = 0.323) and age (*p* = 0.681) did not show additional significant effects and did not significantly improve the model. The association between PAV and the time to perform five repetitive chair rises was r = 0.07 (Spearman). The association between PAV and habitual gait speed was r = 0.37 (Spearman).

The typical error of PAV measured by the activity monitor was 15.3 (95% CI: 11.7; 22.4)°/s, while the SWC_0.2_ was 5.3°/s, and the SWC_0.6_ was 16.0°/s. The Intra-Class Coefficient of agreement describing the reliability between Trials 2 and 3 was ICC_(A,1)_ = 0.76 with a corresponding Pearson correlation coefficient of r = 0.75.

(4) $$\mathrm{PP}\;\left[W\right]=3.91\;\frac{W}{\frac{{}^{\circ}}{s}}\cdot \;\mathrm{PAV}+719.9\;\frac{W}{m}\cdot H-1276.8\;W$$

with PP = peak power [W], PAV = peak angular velocity [°/s], and H = body height [m].

## 4. Discussion

In this study, PAV as measured by a thigh-worn activity monitor showed good agreement with a gold-standard measure and was highly associated with STS power. Furthermore, this measure demonstrated high reliability.

Compared to the PAV values of Pickford et al. [16], our mean values were higher, probably because we included persons with varying physical performance, such as young healthy persons.

The good result when comparing PAV values as measured by the activity monitor with the opto-electronic system has to be seen against the background of the relatively low sample frequency of the activity monitor (20 Hz). Although the observed association was high, it may have been even higher, if PAV was measured by an activity monitor with a higher sample frequency. Here, it is a tradeoff between high accuracy (i.e., high sample frequency) and relatively short measurement duration, due to a higher amount of stored data and a higher energy consumption, and acceptable measurement accuracy (i.e., low sample frequency) but long duration of measurement. Since long duration daily life measurement is aimed for observational studies, the measurement with low sample frequency is preferable. Another advantage of this simple 3-axial acceleration sensor is its high dependability in data acquisition. In contrast, not all STS events could be analyzed by the highly complex force plate/opto-electronic measurement system due to technical problems.

Since a wearable activity monitor was used for data recording, the proposed measure of PAV can be used to assess the quality of single STS transfers during daily life. Here, several STS transfers were recorded during activity monitoring covering hours or days. Therefore, additional parameters, such as variability or minimum and maximum performance, can be used for interpretation. Regarding gait analysis, such in-depth parameters have shown to be relevant for the interpretation of physical activity monitoring [30].

Although in a recent study PAV was able to discriminate between older healthy persons and stroke survivors [16], the construct of this measure remained unclear. According to the concept of the smallest worthwhile change, PAV measured by the activity monitor seems to be able to detect moderate effects (TEM < SWC0.6) [30]. The high association between PAV and peak power in our study demonstrates PAV as a surrogate parameter describing STS power. Since STS power is associated with thigh muscle volume [22], future studies should evaluate STS PAV during daily life in the context of sarcopenia [3].

The weak association of PAV with 5 chair rise time and gait speed can be explained by different instructions to perform the chair rises (as fast as possible versus comfortable pace) and different movements (walking versus chair rise), respectively.

Although the small sample size is a limitation of the study, this aspect was addressed by including persons with a wide range of physical performance, such as younger and older healthy persons and geriatric patients. Therefore, this study can be regarded as a proof of concept. The diagnostic and predictive value of the proposed measure have to be shown in future large-scale prospective studies.

In conclusion, in this sample of persons with a wide range of physical performance, PAV as measured by a thigh-worn acceleration sensor was a valid, relevant, and reliable measure of STS performance. Providing an accurate detection of STS transfers during activity monitoring, PAV can be used as a digital biomarker to describe single STS performance and mean, range, and variability of STS performance during daily life.

## Figures and Tables

**Table 1:** Characteristics of all study participants (*n* = 20).

	Mean (95% CI)	Minimum–Maximum
Age [years]	55.5 (46.4; 64.6)	25–92
Body weight [kg]	71.2 (66.6; 75.8)	57–91
Body height [cm]	172.5 (168.4; 176.5)	1.53–1.85
Body mass index [kg/m^2^]	23.9 (22.8; 25.0)	20.2–28.4
Comorbidity index (0–18)	1.15 (0.67; 1.63)	0–3
Habitual gait speed [m/s]	1.58 (1.41; 1.75)	0.75–2.29

CI = confidence interval; note: the best comorbidity score is underlined.

**Table 2:** Outcome parameters as measured by a thigh-worn activity monitor (peak angular velocity), force plates, and an opto-electronic system (peak angular velocity and peak power) and a stopwatch (chair rise time and gait speed) for all participants (*n* = 20).

	Mean (95% CI)	Minimum–Maximum
Peak angular velocity, 2. trial AM [°/s]	124.6 (110.5; 138.6)	36.0–184.3
Peak angular velocity, 3. trial AM [°/s]	121.7 (108.9; 134.6)	53.6–181.6
Peak angular velocity, 2. trial OES [°/s] *	124.8 (107.3; 142.2)	72.3–176.4
Peak angular velocity, 3. trial OES [°/s] *	121.3 (104.5; 138.1)	63.5–187.5
Peak power, 2. trial FPOES [W] *	465.3 (393.1; 537.5)	295.1–743.2
Peak power, 3. trial FPOES [W] *	452.4 (369.8; 535.1)	224.8–843.9
Five chair rise time [s]	8.4 (7.4; 9.4)	5.2–15.9
Habitual gait speed [m/s]	1.58 (1.41; 1.75)	0.75–2.29

CI = confidence interval; AM = measured by activity monitor; OES = measured by opto-electronic system; FPOES = measured by force plate and opto-electronic system; * peak angular velocity and peak power were not available in 8 measurements (*n* = 4, second trial; *n* = 4 third trial) due to technical problems.

## Data Availability

Data are available on request from the authors.
